# Study on a Mechanical Semi-Active Heave Compensation System of Drill String for Use on Floating Drilling Platform

**DOI:** 10.1371/journal.pone.0133026

**Published:** 2015-07-17

**Authors:** Qingyou Liu, Yang Tang, Chongjun Huang, Chong Xie

**Affiliations:** 1 School of Mechatronic Engineering, Southwest Petroleum University, Chengdu, China; 2 State Key Laboratory of Oil and Gas Reservoir Geology and Exploitation, Southwest Petroleum University, Chengdu, China; 3 Xi Hua University, Chengdu, China; 4 Drilling & Production Engineering Technology Research Institute, Chuanqing Drilling Engineering Company Limited, CNPC, Guanghan, China; University of California Berkeley, UNITED STATES

## Abstract

There are some disadvantages for existing heave compensation systems of drill string used for the Floating Drilling Platform (FDP), including high energy consumption, large and complex structure, and expensive manufacturing and maintenance costs. In view of the above, we present a streamlined mechanical semi-active heave compensation system (MSAHC) in this study. This system consists of active compensation part with the pinion and rack and passive compensation part. In order to evaluate system performance of the MSAHC, we establish its simulation model with AMEsim software. In the process of simulation, displacement of rotary hook and energy consumption is regarded as performance parameters of the system. And the change rule of two performance parameters are analyzed by changing these design parameters including gear radius of the pinion and rack, scale coefficient of PID, rotary hook load, heave height and heave period of the FDP, and accumulator volume. Then, based on the simulation results of the MSAHC system performance, we have selected out a best set of design parameters from them. Moreover, the feasibility of the design scheme of the MSAHC is effectively verified by comparison with the existing three heave compensation system. The result shows that the energy consumption of the MSAHC is lower than the active heave compensation system (AHC) and the semi-active heave compensation system (SAHC) when achieving a same compensation effect as well as the accumulator volume of MSAHC is half of the passive heave compensation system (PHC). Therefore, the new designed MSAHC not only ensure compensation effect but also lower energy consumption, and its structure is simplified by adopting the simple mechanical structure to decrease manufacturing cost, maintenance cost and floor space.

## Introduction

To meet the increasing market demand of industrial production and daily life, such as transportation sector and manufacturing industry, the development of oil and gas has turned from the shallow seas to the deepwater gradually [1–3]. In the exploration and development of deepwater oil and gas, the FDP will come up heave, roll, pitch and yaw influenced by the wind, wave and ocean current [4]. The heave motion of the FDP can bring about the drill string in the process of drilling to perform a reciprocating move up and down. That will lead to drilling weight change on drill bit and even make the drill bit break away from bottom hole. And then drilling efficiency is reduced, service life of the drill string and the drill bit is decreased, and even drilling safety problems may be given rise [5] [6]. Therefore, it is necessary to add some appropriate compensation measures to eliminate the reciprocating heave movement of drill string in the offshore drilling process. By investigation found that the heave compensation system of drill string on the FDP is one common way to solve the problem above at present [7] [8].

The existing heave compensation systems used on the FDP mainly have three different concepts, namely passive heave compensation; active heave compensation and semi-active heave compensation [9]. Over the years, a lot of research works on three heave compensation systems have been done, through a variety of measures including scheme design, theoretical analysis, simulation and optimization, etc. Recent research on PHC systems include the work by Driscoll et al.[10] who found stiffness and damping characteristics of a passive cage-mounted heave compensator, and by Jia et al. [11] [12] who proposed a PHC system with accumulators and carried out a simulation and modeling research. Another work on AHC is that Hao and Liu et al. [13] who presented a kind of AHC system design scheme which the control object was variable pump and electro-hydraulic proportional directional valve, and Xu et al. [14] proposed a principle and mathematical modeling of a new AHC system. Moreover, a new SHAC system has been developed lately by Zhang et al. [15] and model and simulation of the system were also done to ensure necessary design performance. In order to solve the conflict between compensation effect and power consumption of heave compensation system, a new type of drill string heave compensation system is designed by H. Jiang et al. [16].

However, the previous research for the heave compensation systems had failed to consider these problems associated with high energy consumption, large and complex structure, expensive manufacturing and maintenance cost and big floor space and so on. In order to achieve energy conservation, cost reduction and environment protection, it is necessary to carry out an improvement and optimization for the heave compensation system to make up for the above deficiencies. Therefore, we designed a new heave compensation system of drill string——MSAHC in this paper.

The remainder of this paper is organized as follows: Section Establishment of Design Scheme, the active compensation part of the system was designed by combining the advantages of mechanical structure, and passive compensation part was improved based on the existing SAHC system. The Establishment of Simulation Model introduces the mathematical model of main components and the simulation model of the MSAHC established based on the AMEsim software. In Section Results and Discussion, the change rule of the system performances were analyzed by setting different design parameters and a comparative analysis of the existing heave compensation systems was performed. Finally, Section Conclusion summarizes our work and future research avenues.

## Methods

### Establishment of Design Scheme

#### Design for Active Compensation Part of the MSAHC

The whole design scheme of the MSAHC consists of active compensation part and passive compensation part. In order to simplify the system structure to reduce the manufacturing and maintenance cost, the active compensation part was considered using a simpler mechanical structure. The dominating function of the mechanism is to drive piston rod of the MSAHC to move up and down along a straight line [17]. Through massive information acquisition of conventional mechanism, the drive mechanisms used to achieve linear motion were listed as shown in Table 1[18].

**Table 1:** Conventional mechanism structure of linear motion.

Institutions	Work characteristics
CAM mechanism	It is high speed movement, compact structure and high reliability. But it is easy to wear and difficult to machine, and its strokemm is small.
Slider-crank mechanism	It is less wear, big transmission power and low cost. But it is restricted by space so that crank diameter can't be too big
Belt drive	It is simple structure, stable transmission and has buffer load and safety protection. But it is low efficiency, short service life and can't turn-back.
Chain drive	It is high efficiency, strong applicability, big transmission power and low cost. But it is easy to wear and has big noise.
Leadscrew and nut mechanism	It is stable transmission, high precision, but machining precision of its long leadscrew is difficult to guarantee and has limited movement distance.
Pinion and rack	It is high performance, simple structure, convenient processing, and high transmission precision and wear-resisting.

According to the comparative analysis of characteristics of every mechanism, the pinion and rack possesses higher performance including simple structure, convenient processing, precision transmission and wear-resisting. So we deemed that the pinion and rack type meet better the design requirements of the system. And we chose the pinion and rack as the active transmission mechanism of the active compensation part.

**Fig 1:**
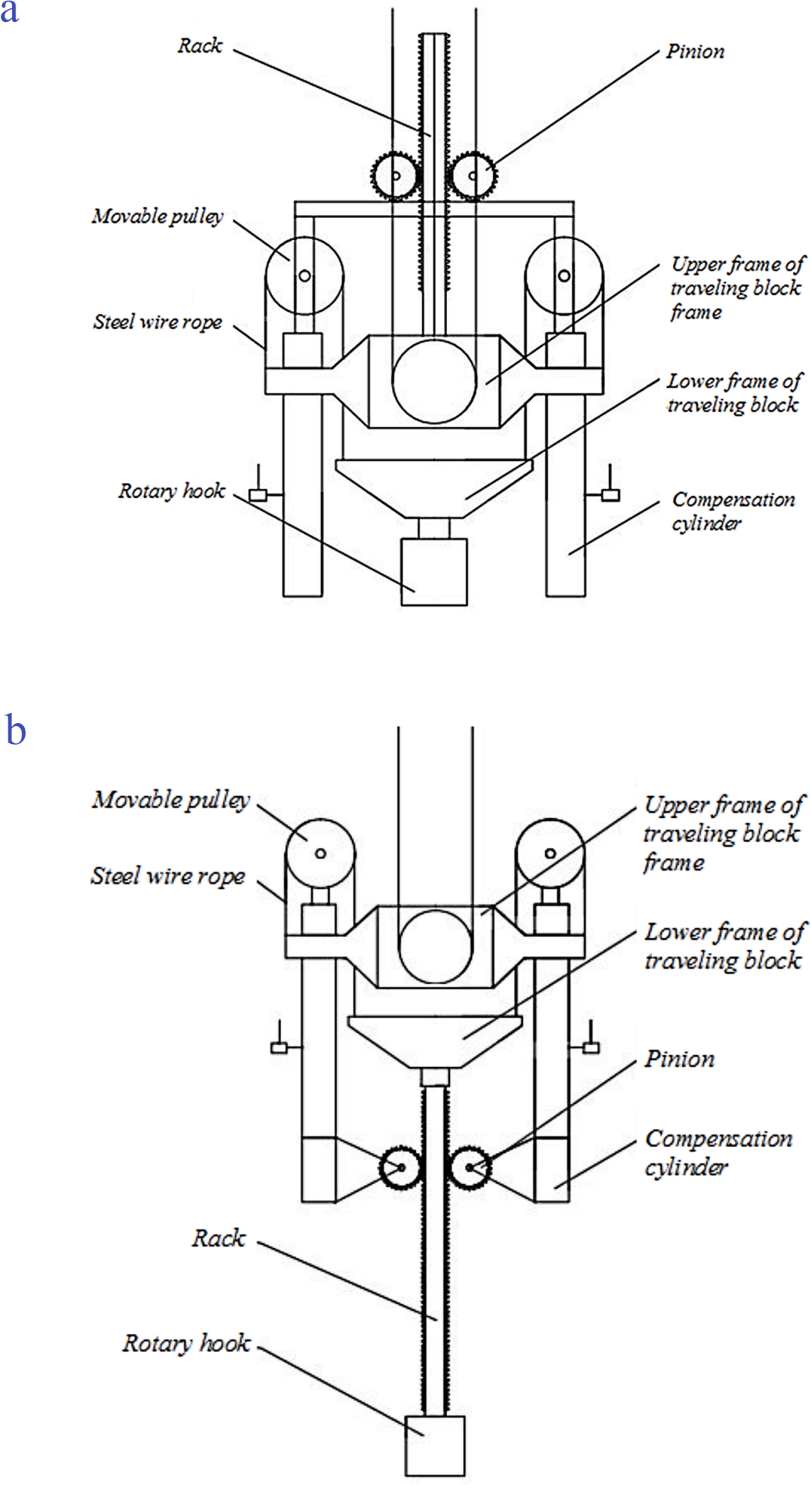
The design of active compensation system with the pinion and rack. (a) Design scheme 1 and (b) Design scheme 2.

Besides, the active compensation part adopted a motor to drive the pinion and rack, and then the system implemented an active compensation by controlling the motor to ensure movement of a piston rod of the system. Based on the pinion and rack, we proposed two design schemes for the active compensation part as shown in Fig 1:

#### Design scheme 1

One **Rack** is fixed on **Upper frame of traveling block** and two **Pinions** are connected to a support plate fixed on two piston rods. When the system starts running, the two **Pinions** are driven by the motor. Then the two **Pinions** and the support plate move up and down along direction of the **Rack**. Finally, a compensation of heave motion is performed without moving the drilling string through the movement of the piston rod with the support plate.

#### Design scheme 2

One **Rack** is fixed on **Lower frame of traveling block.** The bottom of the rack is connected to one **Rotary hook**, and two **Pinions** are installed respectively on the bottom of two **Compensation cylinders** [19]. The working principle of the Design scheme 2 is just like Design scheme 1 except that the **Rack** is driving to move up and down by two **Pinions**.

The comparison analysis on the advantages and disadvantages of these two schemes were analyzed as follows.

#### Design scheme 1

As the pinion and rack is installed on the upper frame of traveling block, it comes true a multiplicative path in running process. Then the displacement of the rack in vertical direction is only half of compensative displacement that the drill string needs. And the whole system structure in the Design scheme 1 is very compact and simple, as shown in the Fig 1A;

#### Design scheme 2

In the Fig 1B, the pinion and rack is installed the lower frame of traveling block, so the displacement of the pinion is equal to the compensation distance. For the Design scheme 2, by contrast, its whole structure is bigger and operating space is also larger. Moreover, the stability of the system is not ensured.

On comprehensive analysis, it was decided that the Design scheme 1 was a more suitable design scheme of the active compensation part.

#### Design for Passive Compensation Part of the MSAHC

**Fig 2:**
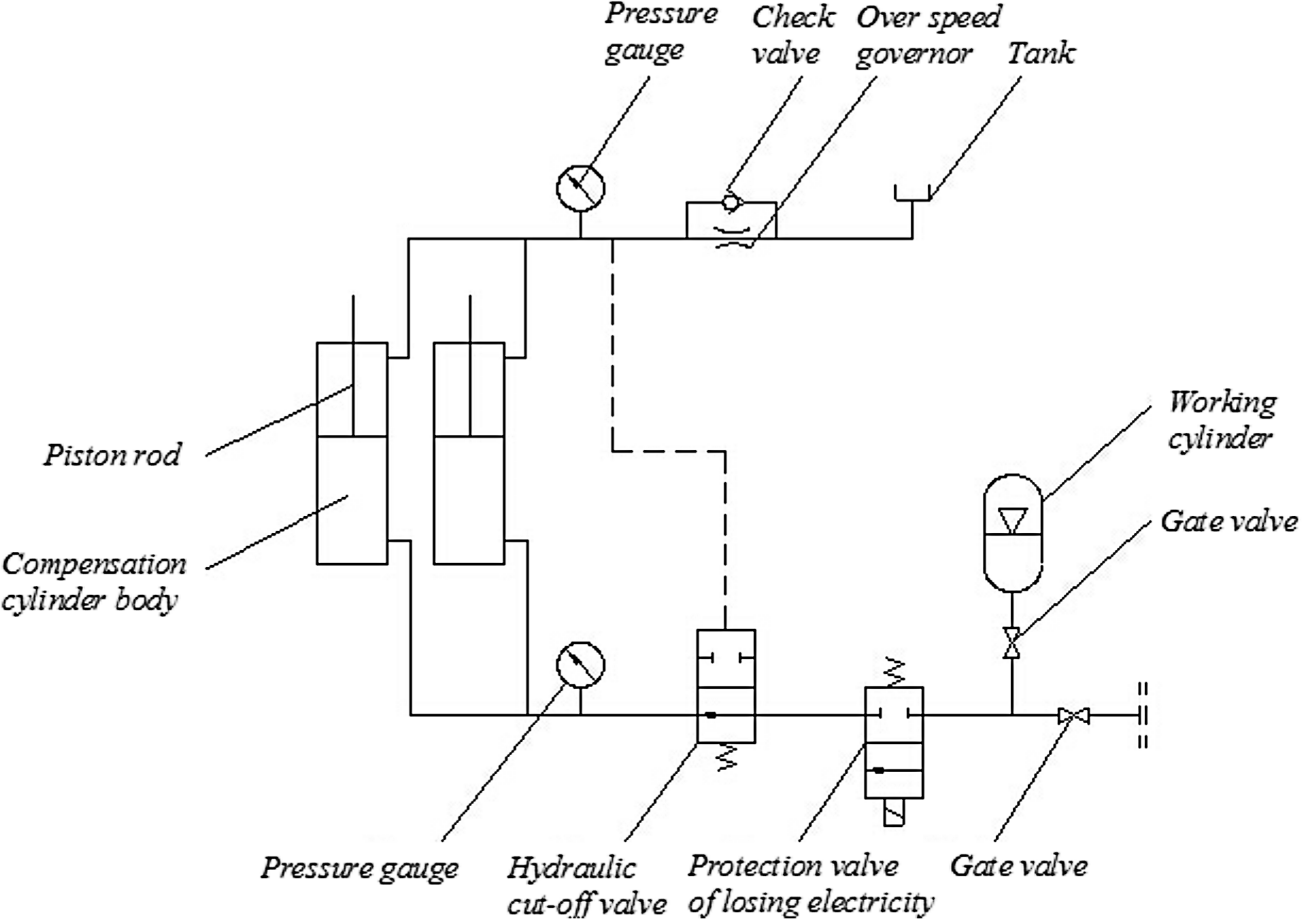
The diagram of passive compensation system.

As shown in Fig 2, the passive compensation part of the MASHC was designed based on the heave compensation strategy of displacement type of the existing SAHC [20]. In the process of the passive compensation of the MASHC, the pressure in **Working cylinder** can be adjusted by making the force of **Piston rod** reach a balance with change of the rotary hook load. In the meantime, the **Piston rod** is staid in one intermediate position of **Compensation cylinder body**. When the rotary hook loads increases, the system will add the work pressure by injecting a high pressure gas to the **Working cylinder**. When the rotary hook load decreases, the work pressure is also lowered by releasing the gas in the **Working cylinder** with exhaust valve. In addition to the conventional function above, the passive compensation system of the MASHC can also be used to adjust the drilling weight on drill bit in drilling process and deal with the accident of drill string broken, etc.

### Establishment of Simulation Model

#### Mathematical Model of the System Components

In the process of establishing the simulation model of the MSAHC with AMESim software, it is necessary to identify movement relationships, logical structures and transmission ways among every component. Through analysis of every component in the MSAHC, we have obtained these mathematical models of main components, including rotary hook load, hydraulic cylinder, pinion and rack, hydraulic pipe, accumulator and PID control system. These mathematical models are directly applicable to the establishment of simulation model of the MSAHC, which given by.

(1) Dynamics model of the rotary hook load [12]

$$M\frac{d^{2}x_{2}}{dt^{2}}=-k_{2}x_{2}+c_{2}\frac{dx_{2}}{dt}+F_{1}+\delta k_{2}-Mg$$ (1)

(2) Mathematical model of the hydraulic cylinder
  - flow continuity equation of the compensation cylinder [14]

$$q_{L}=\frac{K}{V_{c}}\int \;\left(A_{p}v_{L}+\lambda_{c}p_{L}+qL\right)dt$$ (2)

- equilibrium equation of movement of the piston rod

$$M_{h}\frac{d^{2}x_{h}}{dt^{2}}=k_{1}(x_{h}-x_{1})+c_{1}(\frac{dx_{h}}{dt}-\frac{dx_{1}}{dt})+f_{1}-F_{1}-F_{2}$$ (3)

(3) Mathematical model of the pinion and rack [9]

$$T-F_{2}r=mr(\frac{d^{2}x_{h}}{dt^{2}}-\frac{d^{2}x_{1}}{dt^{2}})$$ (4)

(4) Mathematical model of hydraulic line.
  The hydraulic line between the compensation hydraulic cylinder and the accumulator was simplified a vessel. The pressure in it is given by [21–22]:

$$p_{x}=\frac{K}{V_{c1}}\int \;\left(q_{x}+2q_{L}\right)dt$$ (5)

(5) Mathematical model of accumulator
  The ideal gas state equation is defined as follow [21]:

$$p_{sb}V_{sb}^{n}=p_{0}V_{0}^{n}=p_{x}V_{x}^{n}=const$$ (6)

Because the speed of releasing energy is very fast in the process of heave compensation, the hydraulic oil can be considered as an ideal gas working under adiabatic condition. And taking *n* = 1.4. Volume change Δ*V*_*sb*_ of accumulator is given by

$$\Delta V_{sb}=x_{b}A_{p}$$ (7)

Assuming that the gas is in the adiabatic condition and the compressibility of the hydraulic oil is not considered, the accumulator pressure change Δ*p*_*sb*_ is given by

$$\left\{ \begin{array}{l} \Delta p_{sb}={\left(\frac{p_{sb0}V_{sb0}}{V_{sb0}+x_{b}A_{sb}}\right)}^{1.4}-p_{sb0}\\ x_{b}=(x_{2}-x_{1})/2 \end{array}\right.$$ (8)

(6) Movable pulley
  Due to the multiplicative path effect of the movable pulley group, rotary hook displacement *x*_2_ is computed as shown in the following equation:

$$x_{2}=x_{1}-2x_{b}$$ (9)

**Fig 3:**
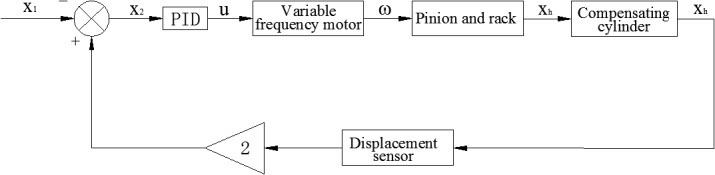
Block diagram of control systems of MSAHC.

(7) Mathematical model of PID
  In the MSAHC, PID is used as the controller by which the motion of the pinion and rack is controlled, and controlling the movement law of the piston in the compensated cylinder is actually realized. The block diagram of control systems of MSAHC was defined as shown in Fig 3, and the control model of PID controller is established as follow [23].

$$u(t)=K_{p}e(t)+K_{i}\int_{0}^{t}e(\tau )d\tau +K_{d}\frac{de(t)}{dt}$$ (10)

#### Establishment of Simulation Model

**Fig 4:**
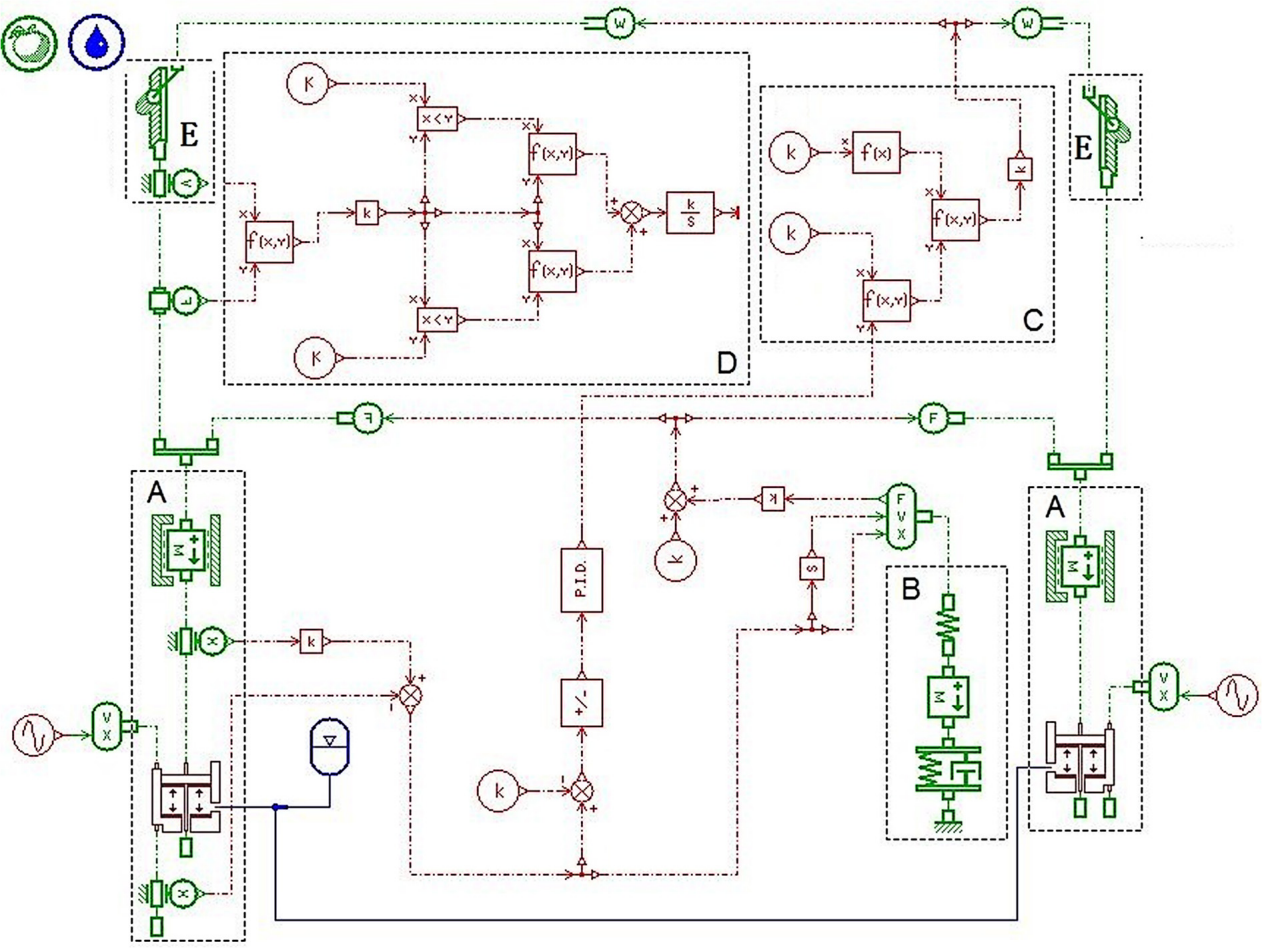
Simulation model of the MSAHC system. Part **A—**The compensation cylinder models; Part **B**—The drill string model; Part **C**—Frequency control of motor speed model; Part **D**–The energy consumption calculation model; Part **E**—The pinion and rack model.

According to the analysis on every unit of the MSAHC, we established its simulation model of the overall system with the AMESim software, as shown in Fig 4 [24]. The simulation model of the MSAHC runs as follows: When the FDP occur a heave motion, the displacement sensor on the compensation cylinder will detect an absolute speed between compensation cylinder and piston rod. And it will be converted into displacement value of the rotary hook which is a relative displacement between the drill string and the FDP. Then the displacement value is input to PID controller after calculation. Furthermore, the PID controller outputs a signal to control motion of the pinion and rack. Finally, the rack pushes the piston in the compensation cylinder to move up or down so that the heave compensate of the drilling platform is achieved.

**Fig 5:**
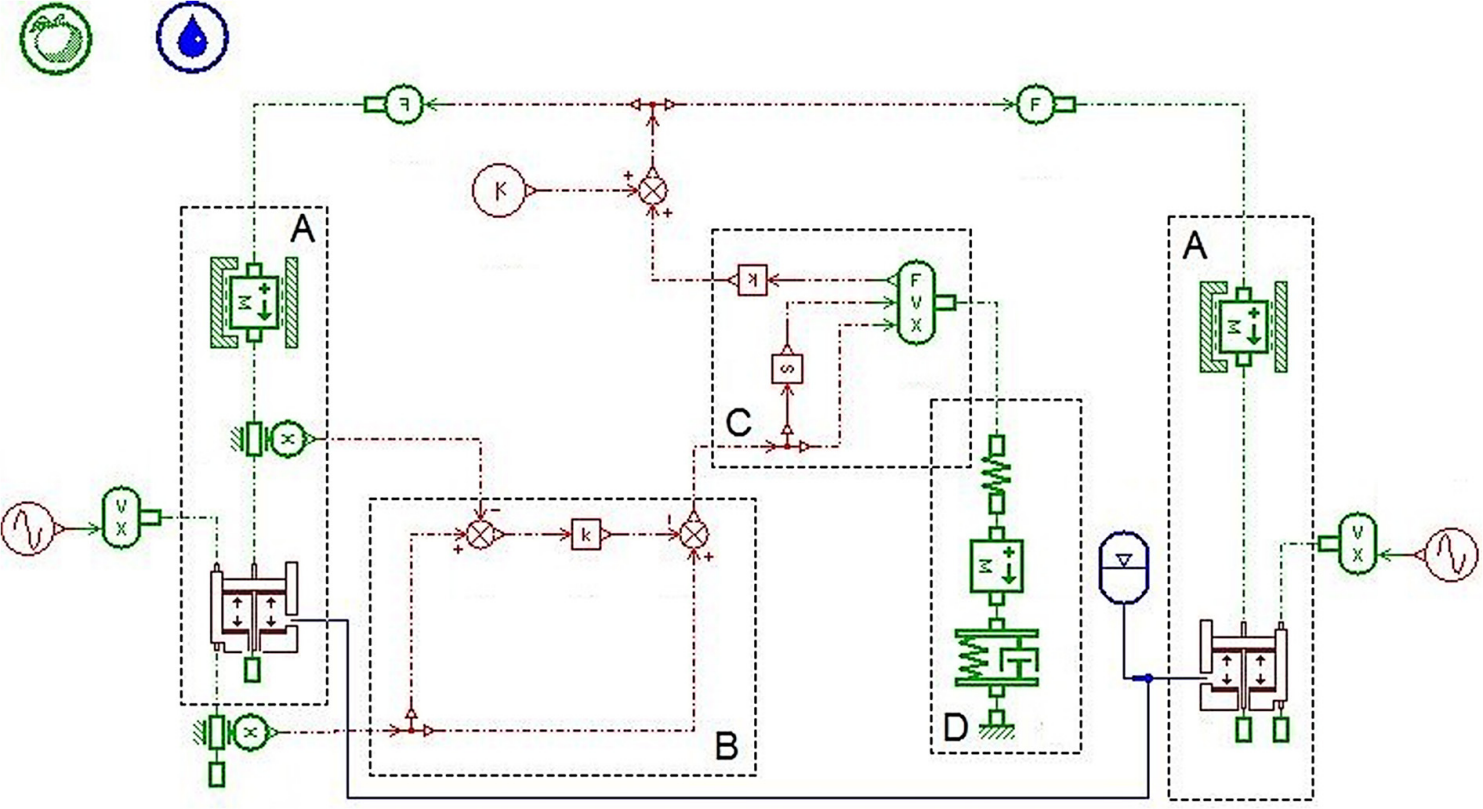
Simulation model of the PHC system. Part **A—**The compensation cylinder model; Part **B**—The rotary hook displacement calculation model; Part **C**—The signal control model; Part **D**–The drill string model;

**Fig 6:**
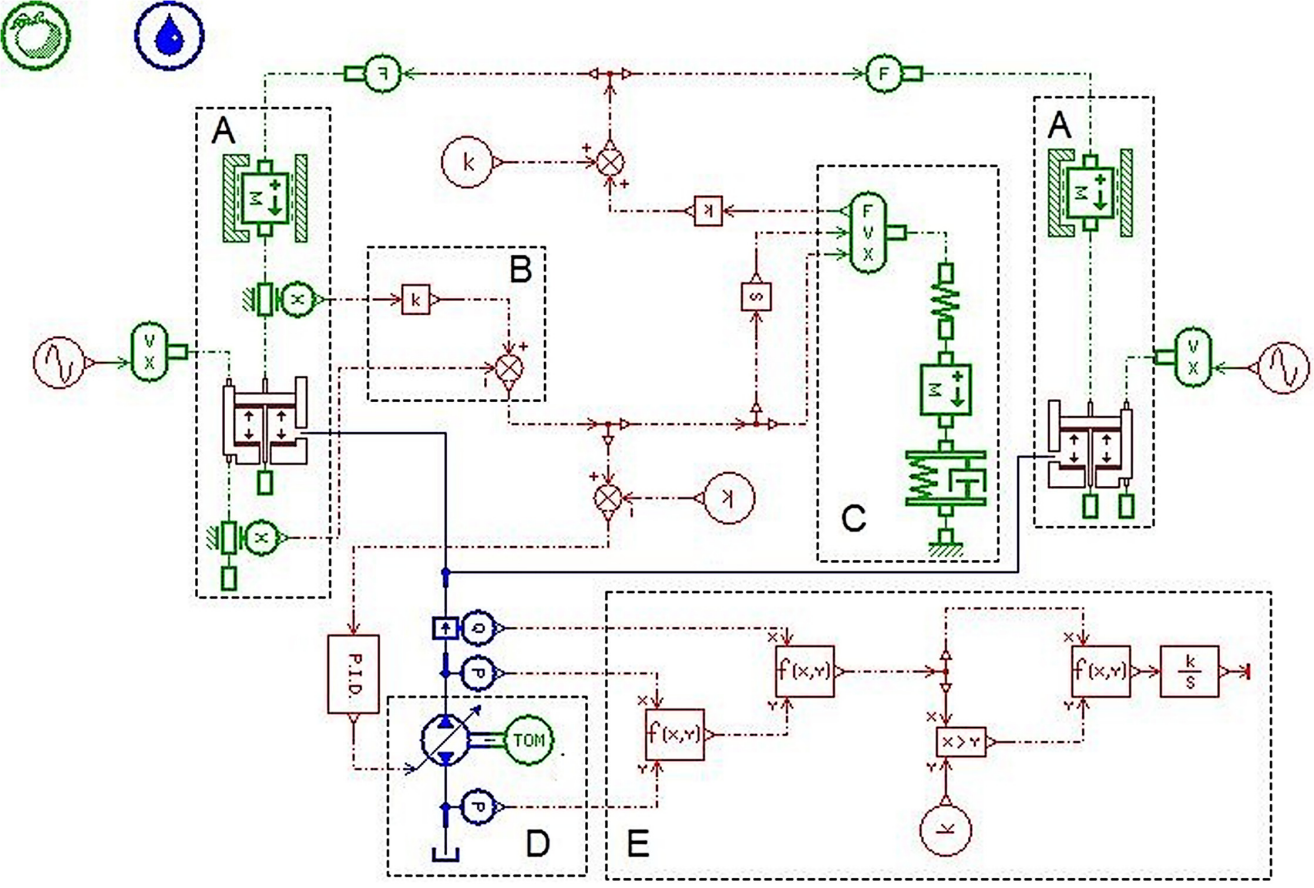
Simulation model of AHC system. Part **A—**The compensation cylinder model; Part **B**—The rotary hook displacement calculation model; Part **C**—The drill string model; Part **D**—The Systems energy supply model; Part **E**—The energy consumption calculation model.

**Fig 7:**
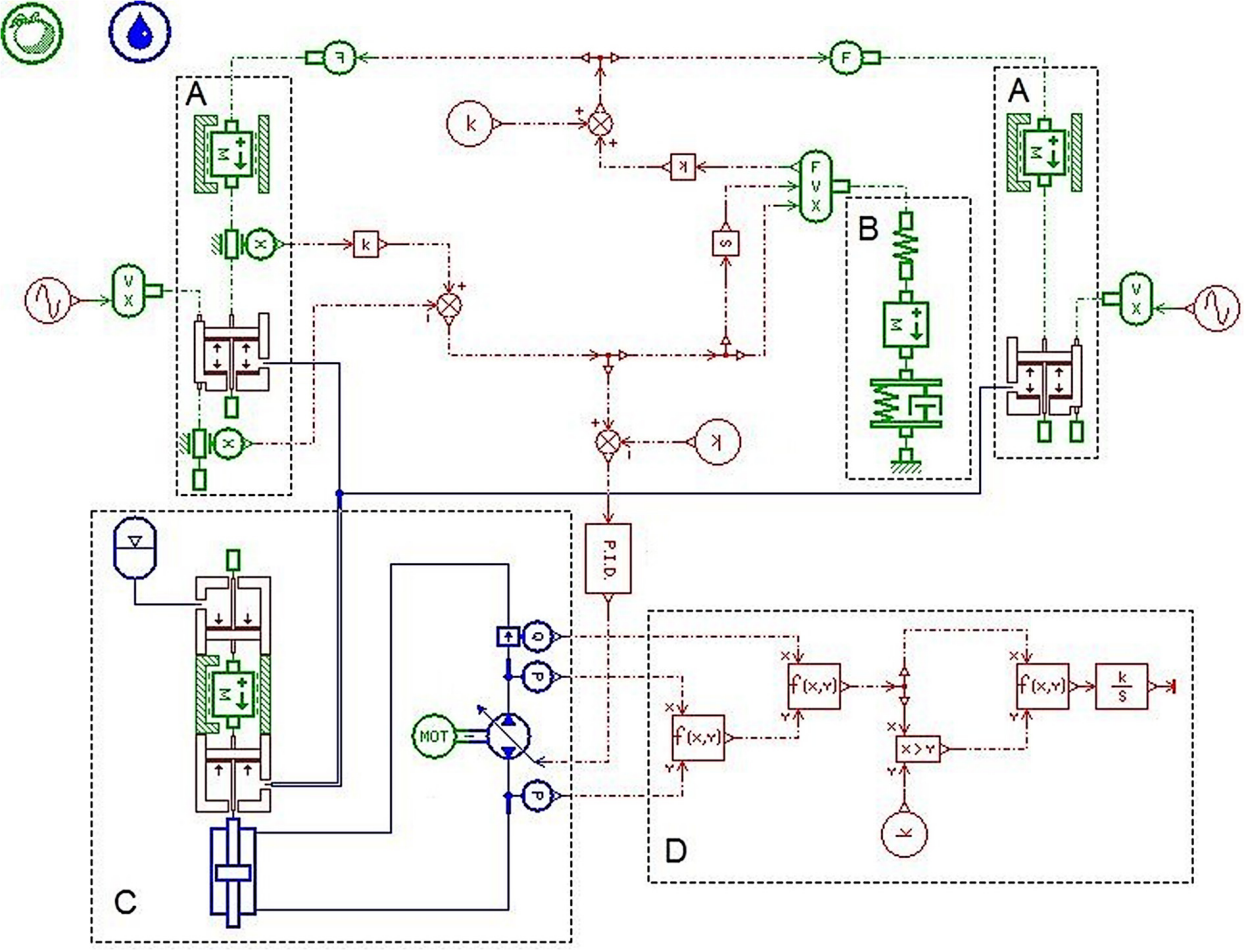
Simulation model of SAHC system. Part **A—**The compensation cylinder models; Part **B**—The drill string model; Part **C**–The compensation system model; Part **D**–The Energy consumption calculation model.

In order to verify usability, desirability and feasibility of the MSAHC, there should be a comparison with other typical heave compensation system including PHC, AHC and SAHC. Based on their working principle diagrams (see S1 Fig, S2 Fig, and S3 Fig), their simulation model were also established by using the AMESim software, as shown in Figs 5, 6 and 7 [12] [14] [15].

## Results and Discussion

### Effects of design parameters on system

In this study, we evaluated the system performance of MSAHC based on the two targets: displacement of rotary hook (DRH) and energy consumption (EC). But they were affected by these design parameters, including gear radius (GR), scale coefficient (SC) and accumulator volume (AV), and environment parameters, including rotary hook load (RHL), heave height (HH) and heave period (HP) of the drilling platform.

#### Effects of GR on system

**Fig 8:**
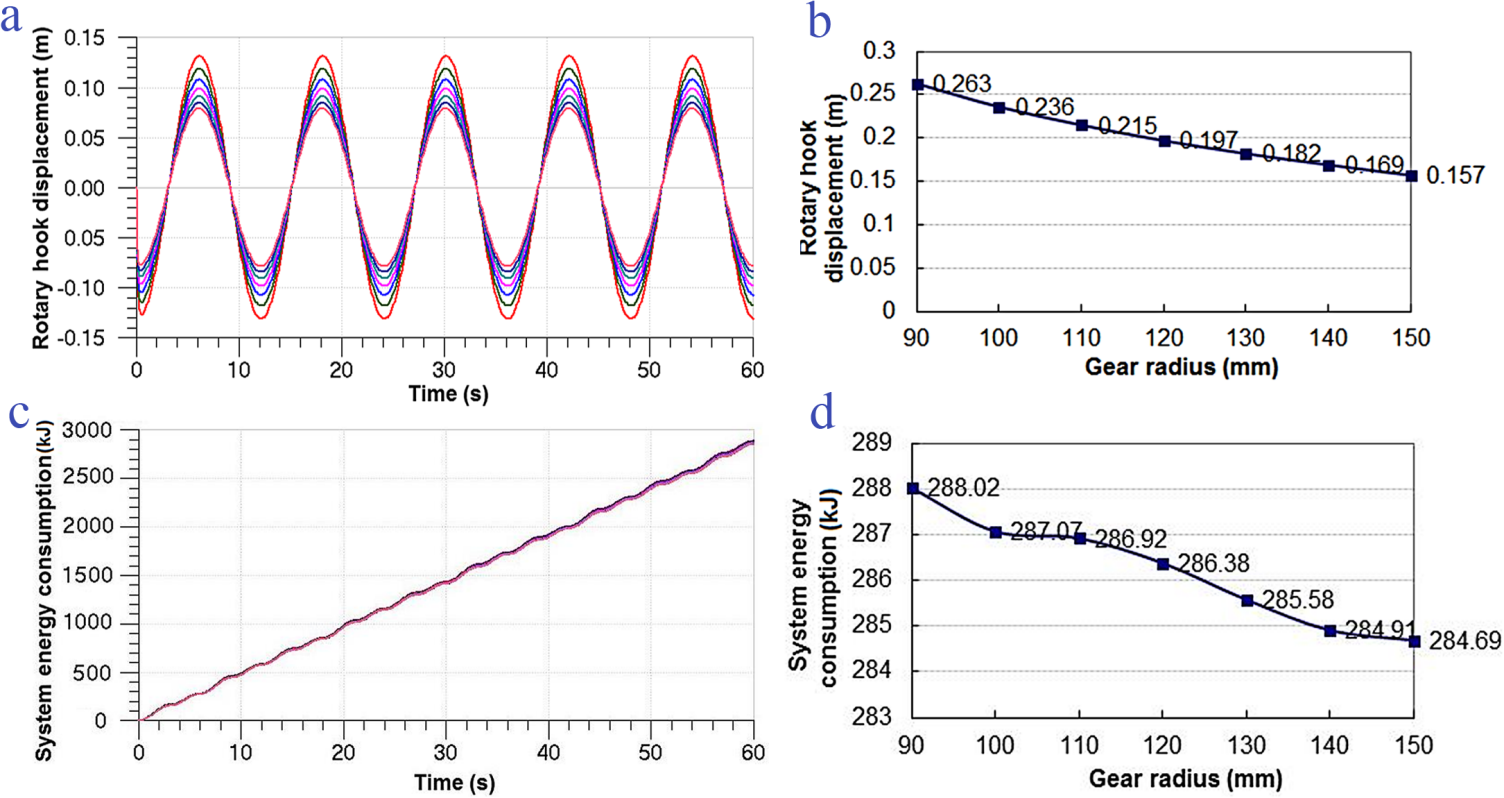
The system performance on different GR. (a) Change rule of the DRH under different GR in 60 seconds, (b) Change rule of total DRH in 60 seconds, (c) Change rule of the EC in 60 seconds,(d) Change rule of total EC in 60 seconds.

By referring to Table 2, system parameters of the MSAHC were set in the simulation model. Then we calculated respectively DRH and EC of the MASHC by taking different gear radius of pinion and rack ranging from 90 mm to 150 mm. The system change rule was obtained with increase of the gear radius and the simulation time in Fig 8. As shown in the Fig 8, it illustrated that the DRH slightly decreased with an increase of gear radius, which means that the compensation effect of the MASHC get more and more better. But there is little change for the EC of the system with increase of the gear radius and the simulation time.

**Table 2:** System parameter settings on different GR.

tParameter	SC	AV	RHL	HH	HP
Value	50	10 *m* ^3^	230t	4.5 *m*	12 *s*

#### Effects of SC on system

**Fig 9:**
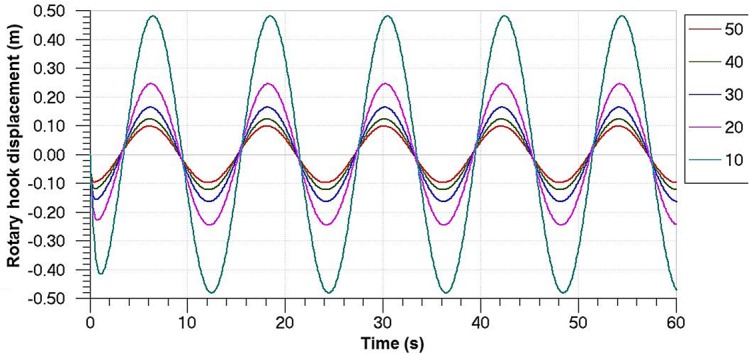
Change rule of the DRH on different SC in 60 seconds.

The system parameters of the MSAHC simulation model were set as shown in Table 3. With the SC of PID controller ranging from 10 to 50, the change rules of DRH in 60 seconds were obtained in Fig 9. It can be seen from the Fig 9 that amplitude of the DRH decreases gradually along with increasing value of the SC, namely the compensation effect of the system became better. Moreover, the system lag is abated effectively because the increase of the SC leads to an increase of output speed of motor. Furthermore, the pinion and rack will be driven to run quickly so that the piston comes back the equilibrium position rapidly.

**Table 3:** System parameter settings on different SC

Parameter	GR	AV	RHL	HH	HP
Value	120 *mm*	10 *m* ^3^	230t	4.5 *m*	12 *s*

**Fig 10:**
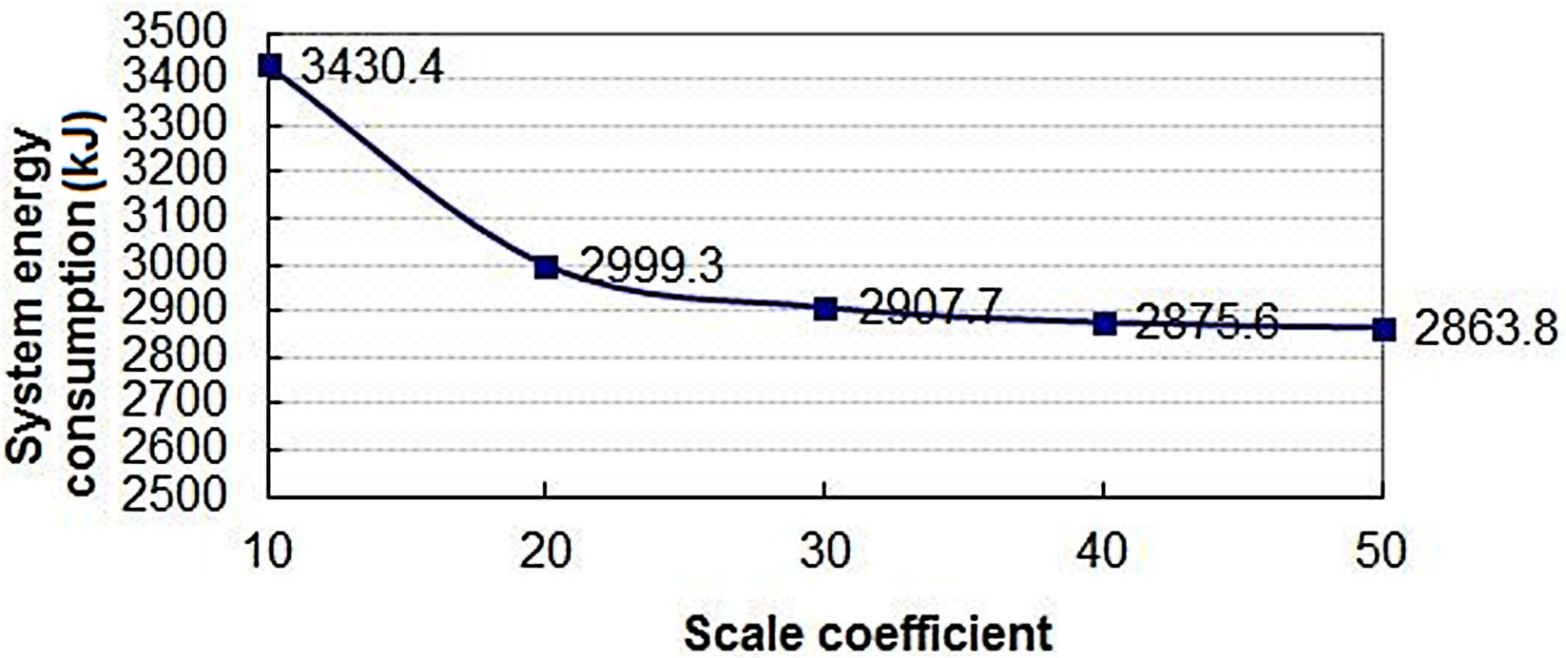
Change rule of total EC in 60 seconds.

Fig 10 shows a cure graph describing the variation trend of the EC with the change of the SC. It is clear from the Fig 10 that the EC is quickly lower when the SC value changes from 10 to 20. But the changes will become obviously slow after reaching 20.

#### Effects of RHL on system

**Fig 11:**
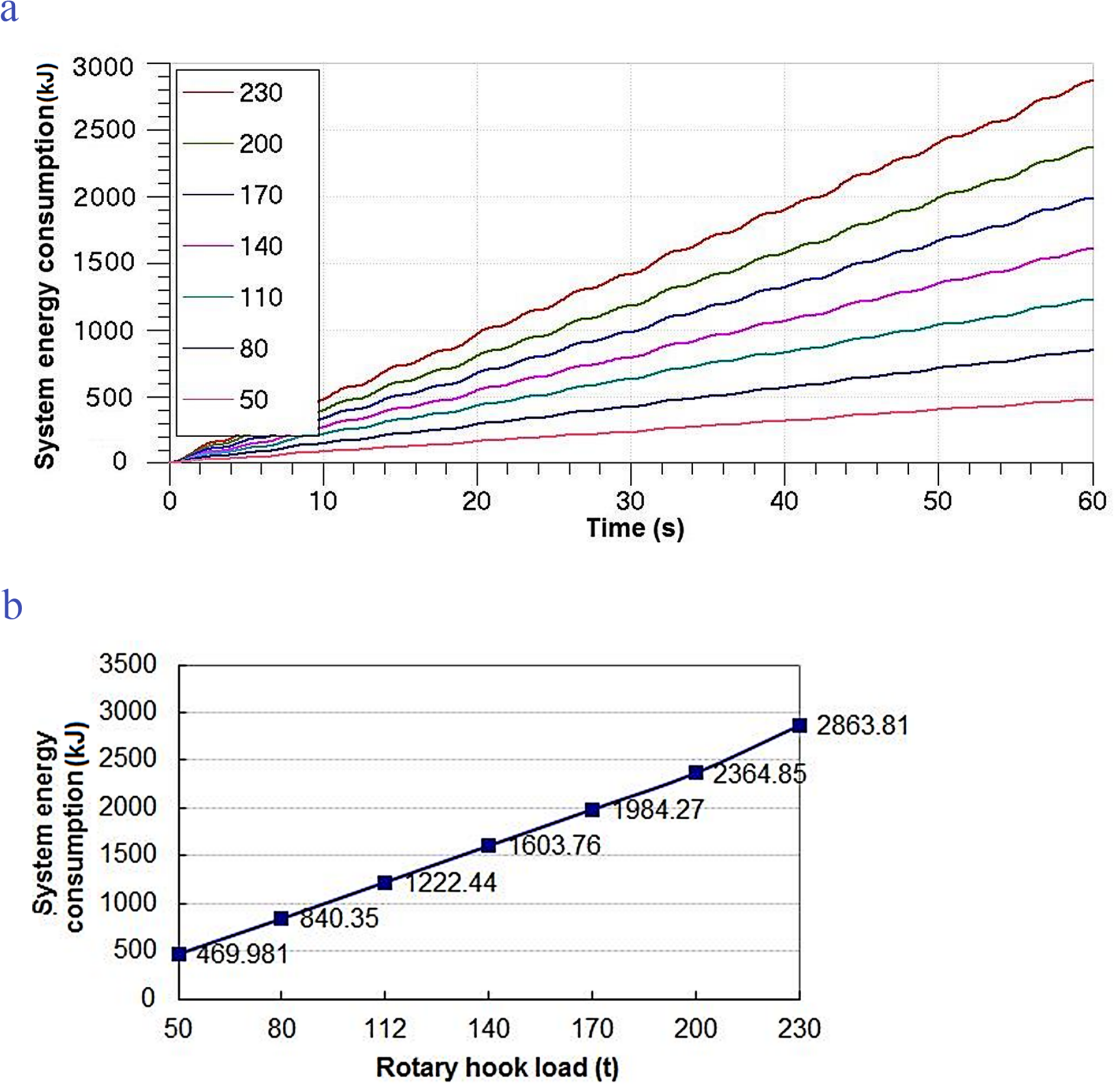
Change rule of the EC on different RHL. (a) Change rule of the EC in 60 seconds, (b) Change rule of total EC in 60 seconds.

With increasing the RHL, a simulation for the MSAHC was carried out by setting system parameters in Table 4. It was found that there wasn’t any change on the compensation effect, but the EC of the system was affected. In Fig 11, the change rules of the EC on different rotary hook load were obtained. From the change rule of the EC in the Fig 11A, we can conclude that a higher rotary hook load will bring about a higher EC. And the Fig 11B shows that the increase rule between the RHL and the EC is an approximate linear proportional relationship.

**Table 4:** System parameter settings on different RHL.

Parameter	GR	SC	AV	HH	HP
Value	120 *mm*	50	10 *m* ^3^	4.5 *m*	12 *s*

#### Effects of drilling platform heave on system

**Fig 12:**
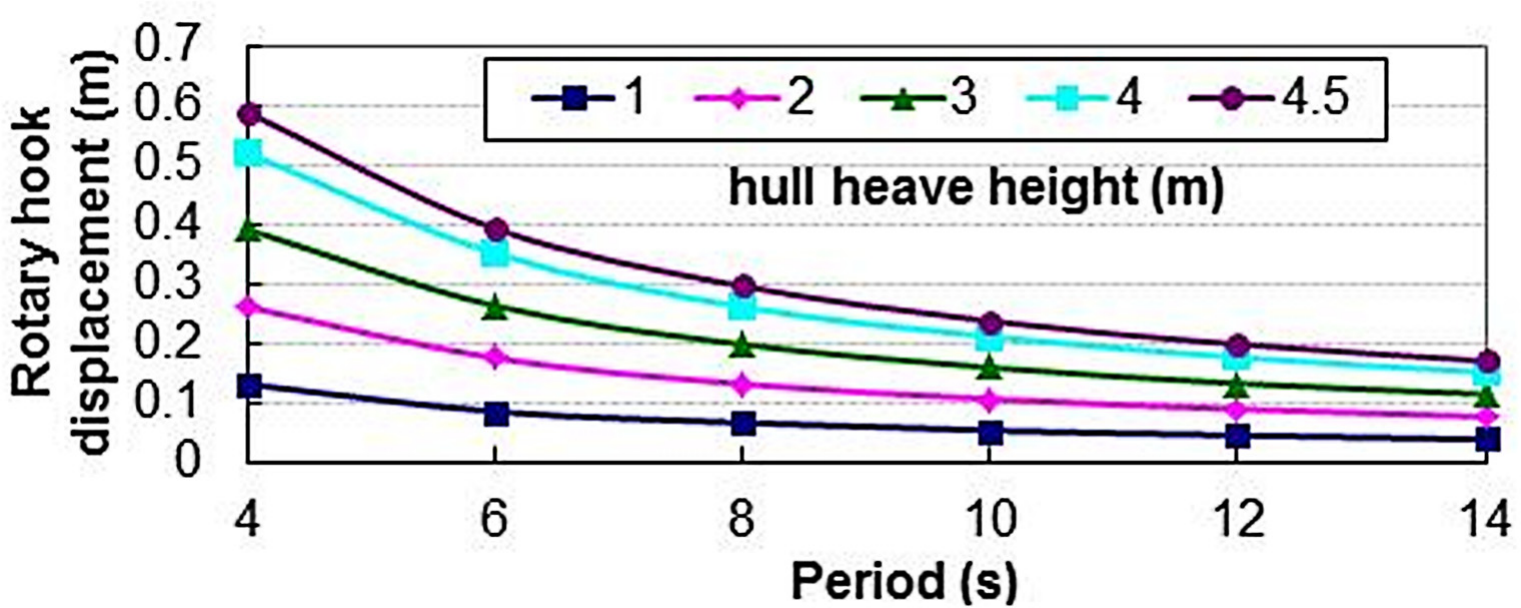
Change rule of the DRH on different HH and HP.

Through a calculation based on system parameters in Table 5, we obtained the change rules of the DRH with the change of the HH and the HP, as shown in Fig 12. It can be concluded from the Fig 12 that the DRH increases gradually with an increase of the HH so that the compensation effect of the system goes down. But with an increase of the HP of drilling platform, the DRH decreases gradually to make the compensation effect better.

**Table 5:** System parameter settings on different HH and HP.

Parameter	GR	SC	AV	RHL
Value	120 *mm*	50	10 *m* ^3^	230t

**Fig 13:**
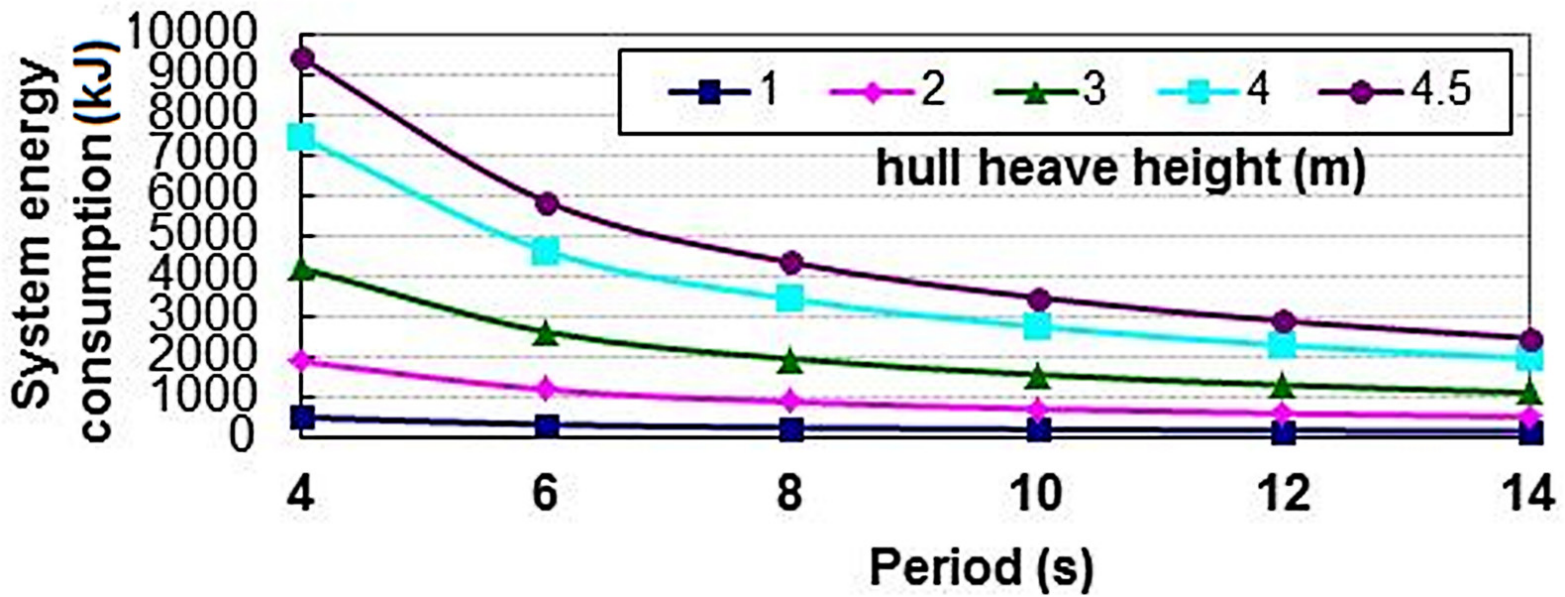
Change rule the EC on different HH and HP.

Moreover, the change rules of the EC were also obtained on different drilling platform heave in Fig 13. It is observed that, for the same HP, the EC of the system increases with an increase of the HH of the drilling platform. And for the same HH, the compensation effect of the system goes down and the EC decreases with a decrease of the HP in a single period.

#### Effect of AV on system

**Fig 14:**
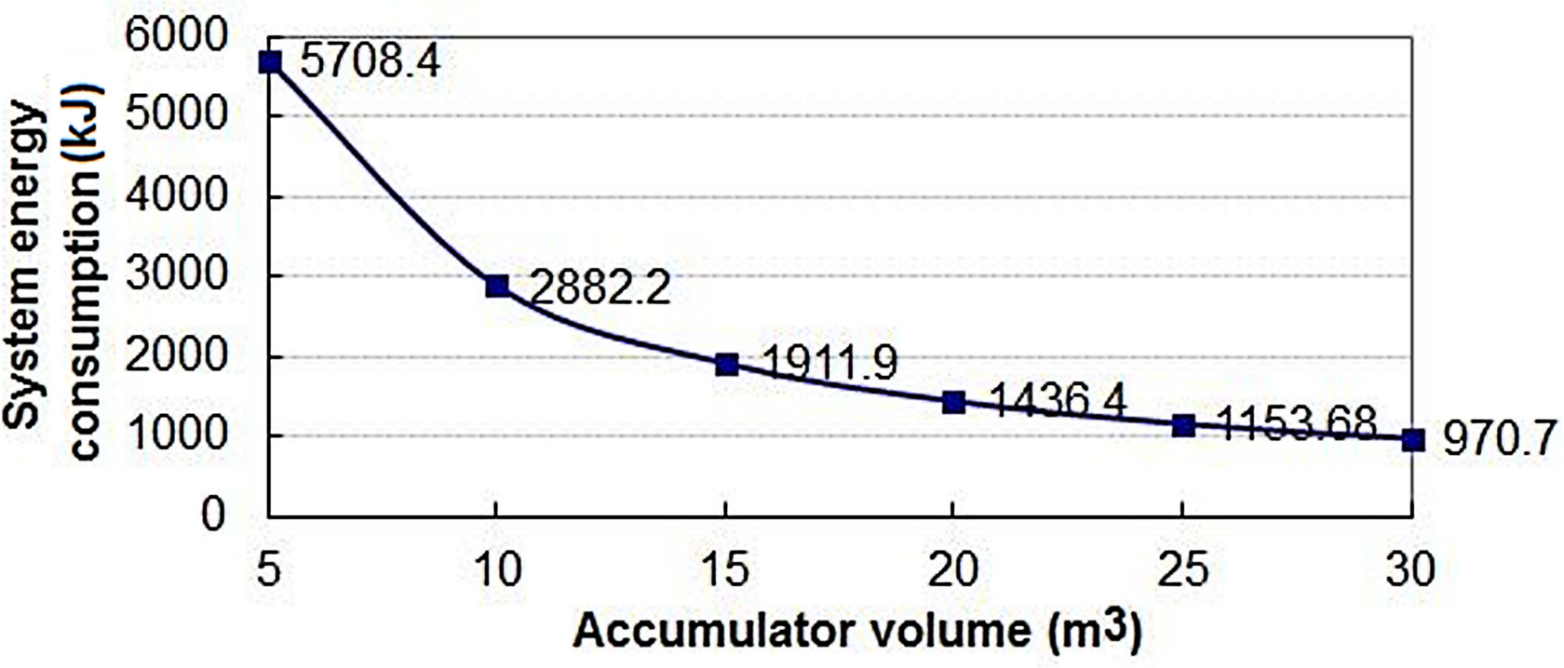
Change of the EC rule on different AV.

By setting the system simulation parameters in Table 6, we obtained Fig 14 which shows change rule of the EC of the system on different AV in 60 seconds. In the Fig 14, the EC continuously decreases with an increase of the AV in the case of other parameters no change. But the change amplitude of the EC is a trend from fast to slow.

**Table 6:** System parameter settings on different AV.

Parameter	GR	SC	RHL	HH	HP
Value	120 *mm*	50	230t	4.5 *m*	12 *s*

### Comparative Analysis of the Four Systems

Based on the simulation models of four systems (PHC, AHC, SAHC and MSAHC) in the Figs 4, 5, 6 and 7, the simulation parameter values were set based on Table 7. And then a lot of simulation analyses on their compensation effect and energy consumption were performed respectively. Finally, their system performances were contrasted in each other to verify usability, desirability and feasibility of the MSAHC.

**Table 7:** Simulation parameters values.

System	Heave period	Heave height	Accumulator volume	Rotary hook load	Scale coefficient	Pump output	Gear radius
PHC	12 *s*	4.5 *m*	20 *m* ^3^	230 *t*	-	-	-
AHC	12 *s*	4.5 *m*		230 *t*	8	12 *L* / *rev*	-
SAHC	12 *s*	4.5 *m*	10 *m* ^3^	230 *t*	2	3 *L* / *rev*	-
MSAHC	12 *s*	4.5 *m*	10 *m* ^3^	230 *t*	50	-	120 *mm*

**Fig 15:**
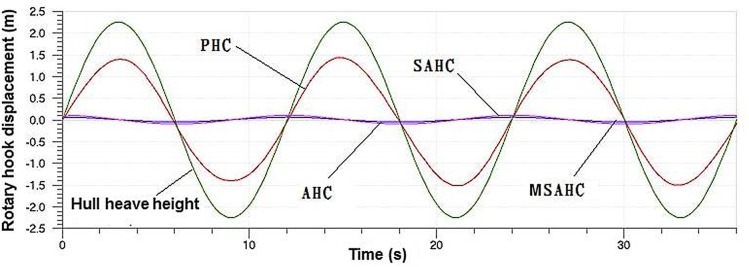
Contrast of heave compensation systems on compensation effect.

Through the simulation calculations, the compensation effects of the four heave compensation systems were shown in Fig 15. From the diagram, we can see that the compensation effect of MSAHC, SAHC and AHC system is little difference, and the displacement fluctuation of the rotary hook using these three systems is within ±75 mm. But the PHC system is poor results about 1.3m deviation.

**Fig 16:**
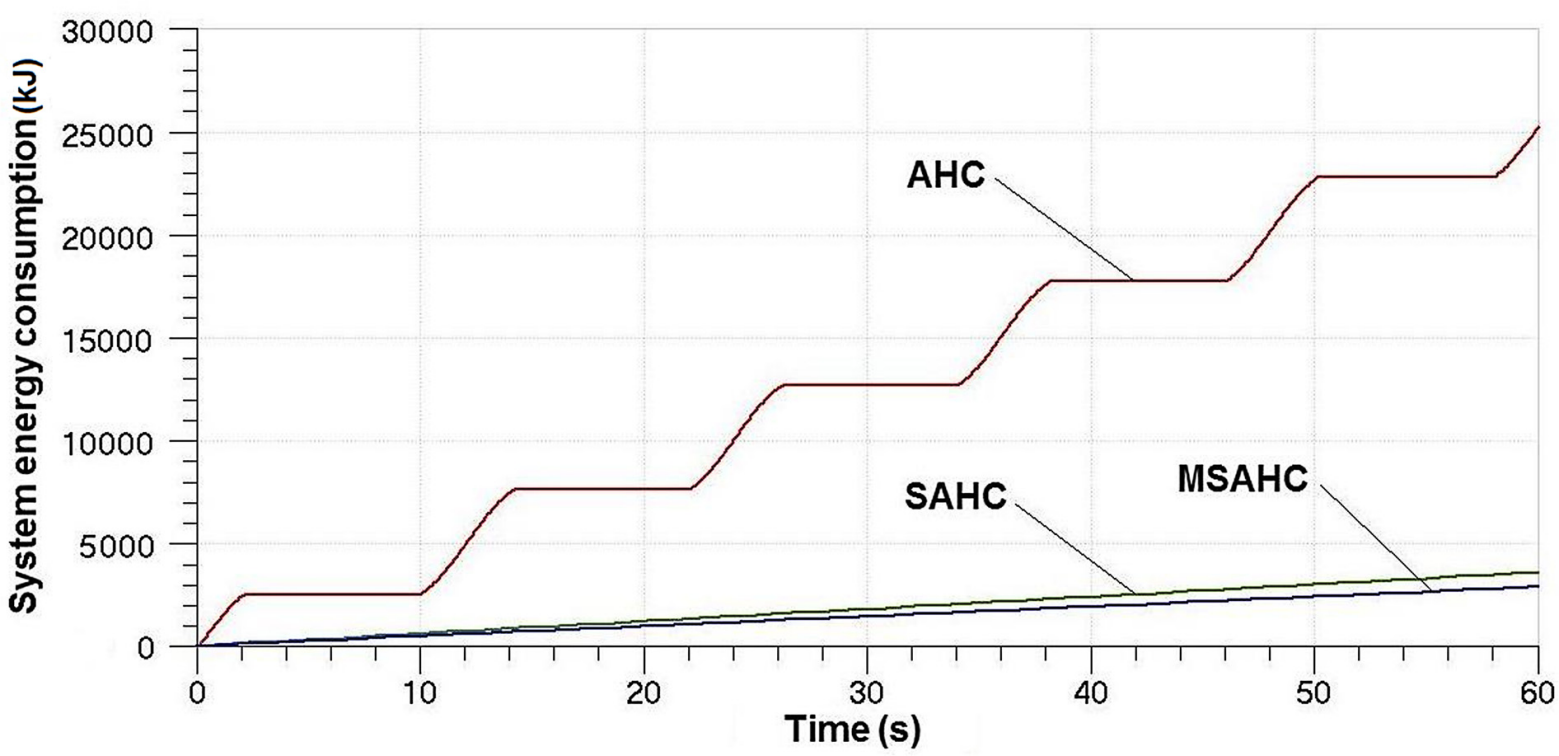
Contrast of heave compensation systems on the EC.

Because there is no energy consumption about the PHC system, we made an analysis on the EC of the AHC, the SAHC and the MSAHC, as shown in Fig 16. Compared with the SAHC and the MSAHC, the EC of the AHC is quite high, which reaches 25000 kJ in the 60 second in the Fig 16. But the EC of the SAHC is about 3572 kJ that is 14.2% of the AHC system. And the MSAHC is 2864 kJ which is lower about 700KJ than the SAHC system. In addition, the accumulator volume of the MSAHC is only equivalent to 50% of the PHC.

In order to ensure drilling operations safety, improve drilling efficiency, and decrease drilling cost, the heave compensation system of drill string is widely adopted on the FDP. For the existing heave compensation system of drill string, there are some problems associated with high energy consumption, large and complex structure, expensive manufacturing and maintenance cost and big floor space. But the previous research on heave compensation system failed to take them into account. So the improvement and optimization for the heave compensation system should be carried out to make up for the above deficiencies. According to structure design and simulation modeling for the MSAHC, we have accomplished the analysis on its design parameters and comparison with three typical heave compensation systems.

From the analysis results of the MSAHC related parameters in the previous section, including gear radius of the pinion and rack, scale coefficient of PID controller, rotary hook load, heave height and heave period of the FDP and accumulator volume, we can draw the following summary:

- Changing the GR of the pinion and rack is only a small effect on the compensation effect and energy consumption of the MSAHC. Therefore, when choosing the gear radius in the design process, we can just consider whether it meet the structural strength and spatial distribution of the pinion and rack.
- To magnify the SC value within allowable scope of the PID controller can significantly improve the compensation effect of the MSAHC and reduce the EC.
- During the drilling process, the EC of the MSAHC has a great relationship with the real-time RHL, and it is an similar proportional growth between them.
- When the drilling platform is a same heave height, the compensation effect of the MSAHC becomes better and the EC is lower with an increase of its heave period. When the drilling platform is same heave period and with enlargement of the heave height, the compensation effect decreases and the EC increases.
- With increasing the accumulator volume, the compensation effect has few change, but the EC quickly lowers and then tends to vary gently.

Based on the comparison with three typical heave compensation systems, the following conclusions were obtained. The compensation effects of the AHC, the SAHC and the MSAHC are much the same in the same working conditions. But the EC of the MSAHC is lower than both the AHC and the SAHC. The MSAHC adopts a simpler mechanical structure to substitute the complex hydraulic structure. So its overall structure become more compact as well as its manufacturing and maintenance cost of the MSAHC are reduced. In addition, the accumulator volume of the MSAHC is only equivalent to 50% of the PHC. So its floor space will can be decreased by almost half.

## Conclusion

In this paper, we have presented a MSAHC of drill string used for the FDP. With the purpose of achieving energy conservation, cost reduction and environment protection, we put forward a new design scheme on its active compensation part. It adopted a simple mechanical structure——the pinion and rack to substituted the existing complex hydraulic structure. And the passive compensation part was improved based on the existing SAHC system. Then the mathematical models of main components and the system simulation model of the MSAHC were established to go to analyze its energy consumption and compensation effect. The change rules of system performances were gained with the change of GR, SC, RHL, HH, HP and AV. Moreover, the contrast results of the four heave compensation systems indicated that the energy consumption of the MSAHC is lower than the AHC and SAHC, and the accumulator volume is half of the PHC. So that it has been basically achieved to lower energy consumption, optimize whole structure, reduce manufacturing and maintenance cost, and decrease floor space. Thus, the feasibility and practicality of the design scheme of the MSAHC is effectively verified. This system is not only that its overall structure is simpler than the existing hydraulic structure to cost savings, but also that its energy consumption is reduced with the precondition of high compensation effect. While these results and data of this study can guide a further detailed design and optimization for the heave compensation system and serve as a reference for subsequent related system research. It should be noted that we do not carry out a prototype machining and some experimental studies of the MSAHC, but these will be our next research work.

## Supporting Information

S1 Fig Working principle diagram of PHC. (TIF)

S2 Fig Working principle diagram of AHC. (TIF)

S3 Fig Working principle diagram of SAHC. (TIF)

## Nomenclature

*A*_*p*_ —Compensation cylinder’s head port area, *m* ^2^

*c*_1_ —Piston resistance coefficient

*c*_2_ —Drag coefficient of drill string

*f*_1_ —Liquid damping force, *N*

*F*_1_ —Rotary hook suffered pull, *N*

*F*_2_ —Rack driving force onto piston, *N*

*k*_1_ —Liquid gas stiffness coefficient

*k*_2_ —Rigid coefficient of drill string, *N*/*m*

*K* —Oil bulk modulus, *MPa*

*K*_*p*_ —Scale coefficient

*K*_*i*_ —Integration time constant

*K*_*d*_ —Derivative time constant

*m* —Quality of the gear, *kg*

*M* —Quality of rotary hook and drill string, *kg*

*M*_*h*_ —Quality of piston and pulley block, *kg*

*q*_*x*_ —Rate of flow of flowing into the accumulator, *L*/*s*

*q*_*L*_ —Rate of flow of flowing into rodless cavity, *L*/*s*

*p*_*L*_ —Liquid pressure of rodless cavity, *MPa*

*p*_*sb*_ —Accumulator charging pressure, *MPa*

*p*_0_ —Initial pressure accumulator, *MPa*

*p*_*sb*0_ —Accumulator stress at the equilibrium position, *MPa*

*r* —Gear radius, *m*

*T* —Motor electromagnetic torque, *N*·*m*

*V*_*c*1_ —Volume of the line vessel, *L*

*V*_*sb*_ —Accumulator aeration volume, *m* ^3^

*V*_0_ —Initial volume of accumulator, *m* ^3^

*V*_*sb*0_ —Volume of accumulator at equilibrium position, *m* ^3^

*x*_1_ —Drilling platform heave displacement, *m*

*x*_2_ —Rotary hook displacement, *m*

*x*_*h*_ —Piston displacement, *m*

*x*_*b*_ —Relative displacement between hydraulic cylinder and piston, *m*

*v*_*L*_ —Relative speed of piston motion, *m*/*s*

*λ*_*c*_ —Leakage coefficient.
