# Supplementary figures and images for: Study on a Mechanical Semi-Active Heave Compensation System of Drill String for Use on Floating Drilling Platform
Source: PLoS One. 2015 Jul 17;10(7):e0133026. doi: 10.1371/journal.pone.0133026 (PMC4505866; doi:10.1371/journal.pone.0133026)

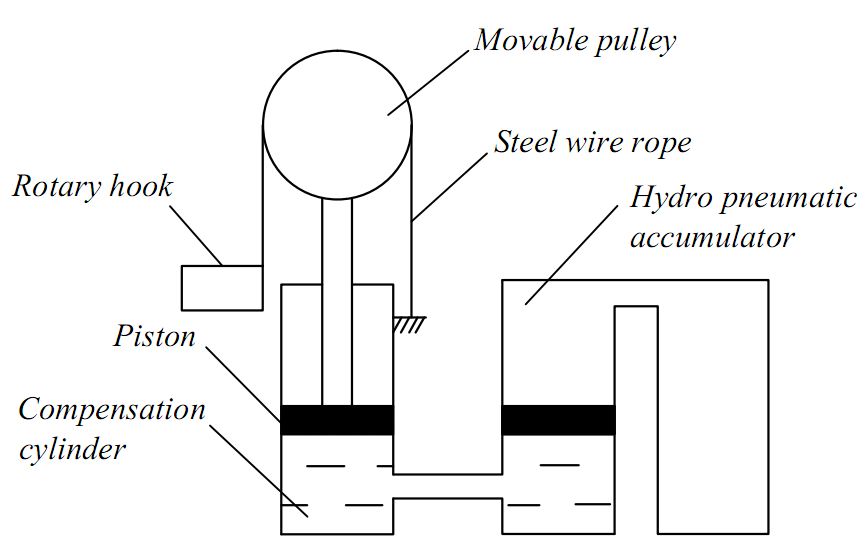

Supplement: S1 Fig — (TIF) [file pone.0133026.s001.tif]

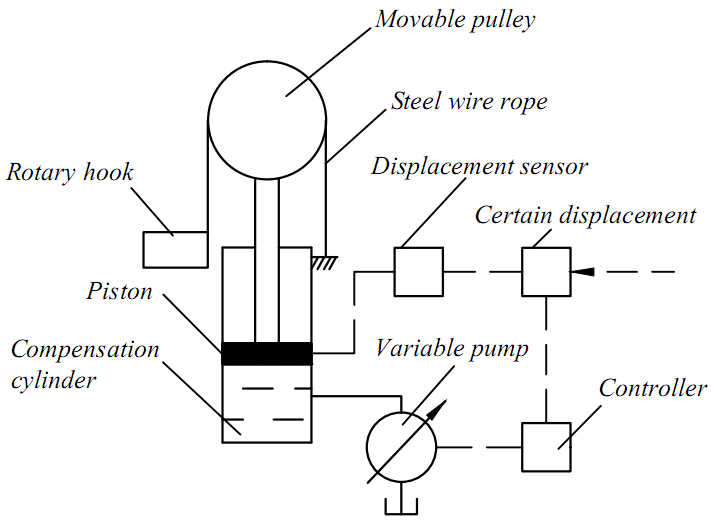

Supplement: S2 Fig — (TIF) [file pone.0133026.s002.tif]

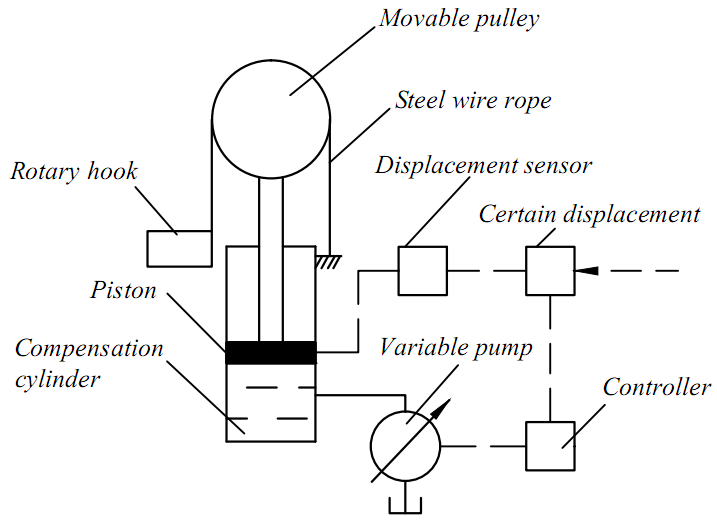

Supplement: S3 Fig — (TIF) [file pone.0133026.s003.tif]
